# Platelet indices and outcome after cardiac arrest

**DOI:** 10.1186/s12873-018-0183-4

**Published:** 2018-09-25

**Authors:** Antonella Cotoia, Federico Franchi, Chiara De Fazio, Jean-Louis Vincent, Jacques Creteur, Fabio Silvio Taccone

**Affiliations:** 1 Department of Intensive Care, Erasme Hospital, Université libre de Bruxelles, Brussels, Belgium; 2 Department of Anaesthesiology and Intensive Care, University of Foggia, Foggia, Italy

**Keywords:** Platelet distribution width, Cardiac arrest, Hypothermia, Platelet indices

## Abstract

**Background:**

Platelet variables, including platelet distribution width (PDW) and mean platelet volume (MPV), have been associated with outcome in critically ill patients. We evaluated these variables in patients after cardiac arrest (CA).

**Methods:**

All adult CA patients admitted to the intensive care unit (ICU) over an 8-year period (2006–2014) and treated with targeted temperature management were included. We retrieved all data concerning CA characteristics as well as platelet count, PDW and MPV on the first 2 days of admission. Unfavorable 3-month neurological outcome was defined as a cerebral performance category score of 3–5.

**Results:**

We included 384 patients (age 62 [52–75] years; 270/384 male): 231 patients (60%) died within 30-days and 246 patients (64%) had an unfavorable 3-month neurological outcome. On admission, platelet count, PDW and MPV were 87 [126–261] *10^3^cells/mm^3^, 17 [16.3–17.3]% and 8.3 [7.6–9.2] μm^3^, respectively. Platelet count decreased significantly over the first 2 days, whereas PDW and MPV did not change significantly. There were no significant differences between the values on admission or time-courses of platelet count, PDW or MPV between survivors and non-survivors or between patients with unfavorable and favorable neurological outcome.

**Conclusions:**

In our cohort of post-CA patients, PDW and MPV were not associated with outcome.

## Background

Despite improvements in the management of patients with cardiac arrest (CA), outcomes remain poor, mainly because of associated cardiogenic shock and post-anoxic brain damage [1]. Moreover, the ischemia/reperfusion injury occurring after the return of spontaneous circulation (ROSC) can contribute to a systemic inflammatory response, similar to that observed in septic patients, which may contribute to organ injury [1–4]. The activation of multiple inflammatory pathways results in platelet activation and an imbalance between endogenous coagulation and fibrinolysis pathways [4, 5]; however, these abnormalities are not easily characterized in clinical practice. In the last few years, there has been increasing interest in platelet variables, such as platelet distribution width (PDW), an indicator of variation in platelet size, and mean platelet volume (MPV), for the diagnosis of various conditions associated with altered platelet counts, including sepsis [6, 7]. These indices, which can be obtained easily from routine blood analysis, have been associated with the occurrence of organ dysfunction during sepsis and an increase in PDW greater than 18% considered as an expression of platelet activation, both being correlated with mortality [8–10].

PDW and MPV have not been widely studied in survivors of CA [11]. It would be interesting to better characterize the prognostic role of these two biomarkers, as they could be used in the future to identify patients with platelet hyperfunction or increased platelet aggregation after CA, who may require for more aggressive antiplatelet therapy, or those with the highest risk of organ dysfunction or systemic reperfusion injury, who might be treated with specific therapeutic approaches.

We therefore investigated the changes in PDW and MPV over time in CA patients treated with targeted temperature management (TTM) and their relationship with outcome.

## Methods

The local Ethical Committee (Comité d’Ethique Hospitalo-Facultaire Erasme-ULB) approved the study (Protocol P2017/264), but the need for informed consent was waived because of the retrospective analysis of data. Non-traumatic patients with Glasgow coma scale < 9, surviving for at least 24 h after in-hospital or out-of-hospital CA and admitted to the intensive care unit (ICU) were included in an institutional database (December 2006 to October 2014) and considered eligible for the study. Exclusion criteria were missing data on blood count on admission.

All patients were treated with TTM (target temperature: 32–34 °C) for 24 h, according to our standardized protocol that has been described elsewhere [12]. Briefly, cooling is started immediately after ICU admission with a combination of cold fluid bolus (20–30 ml/kg of a crystalloid solution in 30 min) and a circulating water blanket device (Medi-Therm II, Gaymar, USA). Target temperature is initially measured using a rectal temperature probe or a blood temperature monitoring in case an invasive haemodynamic monitoring (PiCCO, Pulsion, Munich, Germany) is initiated. Sedation is based on the use of midazolam (0.03–0.1 mg/kg/h) and analgesia on morphine (0.1–0.3 mg/kg/h) infusions. Cisatracurium is administered to control shivering in the induction phase (bolus of 0.15 mg/kg) and, if needed, as a continuous infusion thereafter (1–3 mcg/kg/min). After 24 h of cooling, rewarming is obtained passively at a rate of 0.3–0.5 °C/h and sedation discontinued when body temperature reaches 37 °C. Patients are kept in a 30° semi-recumbent position; ventilation is set to keep PaCO_2_ between 35 and 45 mmHg and SpO_2_ > 94–96%. Blood glucose is kept between 110 and 150 mg/dL and mean arterial pressure maintained > 65–70 mmHg using fluids, dobutamine and/or norepinephrine, as appropriate. Intra-aortic balloon counterpulsation (IABP) or extracorporeal membrane oxygenation (ECMO) are used in cases of severe cardiogenic shock.

In case of persisting deep coma associated with bilateral absence of the N20 response to somatosensory evoked potentials (on day 2–3 after arrest), and/or status myoclonus with malignant EEG (i.e. burst suppression or suppressed background) and/or refractory status epilepticus, decisions to withdraw life-support were made after interdisciplinary discussion and occurred not before 48–72 h after normothermia (> 37°) was restored and sedative agents were stopped.

We collected data on demographics, comorbidities and CA characteristics. The platelet count (normal range: 155–350*10^3^cells/mm^3^), PDW (normal range: 15–18%) and MPV (normal range: 6.4–10 μm^3^) values were obtained on ICU admission and for two subsequent days from the routine daily blood count measured using the UniCel DXH 800 Coulter Cellular Analysis System (Beckman Coulter International S.A., Nyon, France). Thrombopenia was defined as a platelet count of < 150*10^3^cells/mm^3^ while high PDW and MPV in case of values > 18% and 10 μm^3^, respectively. Blood lactate and C-reactive protein (CRP) levels on admission were also recorded. Treatments with vasoactive/inotropic drugs and continuous renal replacement therapy were noted. Shock was defined as the need for vasopressor agents for more than 6 h. The development of infections during the ICU stay was recorded; survival was recorded at 30-days after admission in ICU. Neurological evaluation was assessed using the cerebral performance category score (CPC) during follow-up visits or by telephone interview 3 months after CA [13]. Unfavorable neurological outcome was considered as a CPC 3–5.

### Statistical analysis

Data were analyzed using SPSS 24.0 for Windows (SPSS Inc., Chicago, IL). We used the Shapiro-Wilk test and stratified distribution plots to verify the normality of distribution of continuous variables. Continuous variables are presented as median [25–75 percentile] and number (%) as appropriate. Differences between groups (survivors vs. no survivors, and unfavorable vs. favourable outcome) were assessed using Student’s T test, Mann-Whitney test, χ^2^ test, or Fisher’s exact test, as appropriate. Time-courses of platelet parameters were analyzed using a two-way ANOVA for repeated measurement with Bonferroni *post-hoc* correction. A two-tailed test was performed and a *p* value < 0.05 was considered to be significant.

## Results

From a total of 445 eligible CA patients admitted over the study period, 43 died within a few hours after hospital admission without blood sampling and 18 were excluded for missing PDW/MPV data, leaving 384 patients for the final analysis (mean age: 62 [52–75]; male gender, *n* = 270 [70%] - Table 1). The total duration of ICU stay was 4 [2–8] days; 231 patients died within day 30 (60%) and 246 (64%) patients had an unfavorable outcome.

**Table 1:** Characteristics of study population according to favourable (FO) and unfavourable (UO) neurological outcome

	All (*n* = 384)	Survivors (*n* = 153)	Non-survivors (*n* = 231)	*P* Value	FO (*n* = 138)	UO (*n* = 246)	*P* Value
Age, years	62 [52–75]	58 [49–70]	66 [54–78]	< 0.001	58 [50–69]	66 [53–77]	< 0.001
Weight, kg	77 [67–85]	78 [70–87]	75 [65–85]	0.360	78 [70–88]	75 [65–85]	0.076
Male, n (%)	270 (70)	114 (75)	156 (67)	0.110	103 (75)	167 (68)	0.165
Comorbidities							
Chronic hypertension, n (%)	175 (46)	71 (46)	104 (45)	0.790	63 (46)	112 (45)	0.981
Diabetes, n (%)	95 (25)	33 (22)	62 (26)	0.241	28 (20)	67 (27)	0.130
Chronic heart failure, n (%)	81 (21)	30 (20)	51 (22)	0.561	29 (21)	52 (21)	0.977
Coronary artery disease, n (%)	168 (44)	60 (39)	108 (47)	0.145	56 (40)	112 (45)	0.348
Neurological disease, n (%)	64 (16)	17 (11)	47 (20)	0.028	14 (10)	50 (20)	0.011
Liver cirrhosis, n (%)	21 (6)	4 (3)	17 (7)	0.045	4 (3)	17 (7)	0.097
COPD/asthma, n (%)	66 (17)	23 (15)	43 (18)	0.362	20 (14)	46 (19)	0.294
Presentation rhythm, n (%)							
VF-VT, n (%)	151 (39)	89 (55)	62 (27)	< 0.001	83 (60)	68 (27)	< 0.001
Unknown, n (%)	23 (6)	8 (5)	15 (6)	0.941	6 (4)	17 (7)	0.875
Out of hospital CA, n (%)	215 (56)	84 (55)	131 (57)	0.675	76 (55)	139 (56)	0.739
Time to ROSC, min	15 [7–25]	12 [5–20]	18 [10–25]	0.002	12 [5–20]	17 [10–25]	0.005
Epinephrine, mg	3 [2–6]	2 [1–4]	4 [2–7]	< 0.001	2 [1–4]	4 [2–7]	< 0.001
Witnessed CA, n (%)	323 (84)	138 (90)	185 (80)	0.006	124 (90)	199 (81)	0.021
Bystander CPR, n (%)	255 (66)	138 (90)	117 (50)	0.001	107 (77)	148 (60)	0.001
Cardiac origin of arrest, n (%)	230 (60)	105 (69)	125 (54)	0.004	97 (70)	133 (54)	0.002
During ICU stay							
AKI, n (%)	231 (60)	77 (50)	154 (67)	0.001	69 (50)	162 (66)	0.002
Vasopressor use, n (%)	278 (72)	97 (63)	181 (78)	0.001	88 (64)	190 (77)	0.005
Dobutamine use, n (%)	202 (53)	77 (50)	125 (54)	0.252	69 (50)	133 (54)	0.321
Hypothermia, n (%)	339 (88)	131 (85)	208 (90)	< 0.001	119 (86)	220 (89)	0.435
Sepsis, n (%)	118 (31)	45 (29)	73 (32)	0.649	40 (29)	78 (31)	0.579
IABP, n (%)	28 (7)	8 (5)	20 (8)	0.206	7 (5)	21 (8)	0.210
ECMO, n (%)	53 (14)	20 (13)	33 (14)	0.736	19 (14)	34 (14)	0.988
CRRT, n (%)	60 (16)	25 (16)	35 (15)	0.754	22 (16)	38 (15)	0.898
Shock, n (%)	207 (54)	66 (43)	141 (61)	0.001	61 (44)	146 (59)	0.004
Steroid therapy, n (%)	74 (19)	24 (16)	50 (22)	0.147	23 (17)	51 (21)	0.333
RBC transfusion, n (%)	99 (26)	42 (28)	57 (24)	0.543	37 (27)	62 (25)	0.730
Blood sample on admission							
Creatinine, mg/dL	1.2 [0.9–1.6]	1.1 [0.9–1.6]	1.3 [0.9–1.7]	0.095	1.1 [0.9–1.6]	1.2 [0.9–1.7]	0.082
Lactate, mg/dL	4.4 [2.8–7.9]	3.9 [2.5–5.6]	4.9 [3.1–8.7]	< 0.001	3.9 [2.5–5.7]	4.6 [6.0–8.5]	0.003
CRP, mg/L	11.0 [3.6–53.2]	9.0 [2.9–49.3]	12.5 [4.0–59.0]	0.039	7 [2.3–33.1]	15.1 [4.3–64.5]	0.001
PLT, *10^3^cells/mm^3^	193 [131–266]	196 [133–269]	193 [133–261]	0.654	197 [134–264]	194 [130–268]	0.499
MPV, μm^3^	8.3 [7.6–9.2]	8.4 [7.7–9.2]	8.4 [7.6–9.3]	0.770	8.3 [7.7–9.2]	8.4 [7.6–9.3]	0.864
PCT, %	0.2 [0.1–0.2]	0.2 [0.1–0.2]	0.2 [0.1–0.2]	0.675	0.2 [0–1-0.2]	0.2 [0.1–0.2]	0.774
PDW, %	17.0 [16.3–17.3]	17.0 [16.2–17.3]	17.0 [16.4–17.4]	0.422	16.8 [16.2–17.3]	16.9 [16.4–17.4]	0.628
ICU length of stay, days	4 [2–8]	7 [4–13]	4 [2–7]	< 0.001	6 [4–12]	4 [2–8]	< 0.001

*CA* cardiac arrest, *CPR* cardiopulmonary resuscitation, *ROSC* return of spontaneous circulation, *VF/VT* ventricular fibrillation/ventricular tachycardia, *AKI* acute kidney injury, *ICU* intensive care unit, *IABP* intra-aortic balloon pump counterpulsation, *ECMO* extracorporeal membrane oxygenation, *CRRT* continuous renal replacement therapy, *COPD* chronic obstructive pulmonary disease, *RBC* Red Blood Cells, *MV* mechanical ventilation, *CRP* C-reactive Protein, *PLT* Platelet count, *MPV* Mean platelet volume, *PCT* Plateletcrit, *PDW* Platelet Distribution Width

On admission, the platelet count was 193 [131–266] *10^3^cells/mm^3^, PDW 17.0 [16.3–17.3]% and MPV 8.3 [7.6–9.2] μm^3^ (Table 1). Platelet counts decreased significantly over the next 2 days to 160 [97–215] on day 1 and 147 [87–202] *10^3^cells/mm^3^ on day 2, *p* <  0.001), whereas PDW and MPV remained stable. Thrombopenia was observed in 108 (28%) patients on admission and in 146 (38%) and 203 (53%) on day 1 and 2, respectively. High PDW and MPV were observed in 78 (20%) and 62 (16%) patients on admission, in 80 (21%) and 64 (17%) patients on day 1 and in 82 (21%) and 67 (17%) patients on day 2, respectively.

Compared to survivors, non-survivors were older, had less frequently had a witnessed arrest, bystander CPR, a cardiac origin of the arrest and an initial shockable rhythm, and had longer resuscitation times and higher epinephrine doses (Table 1). They also had higher admission lactate and CRP concentrations, more frequently received vasopressor therapy, and had a shorter ICU length of stay.

**Fig. 1:**
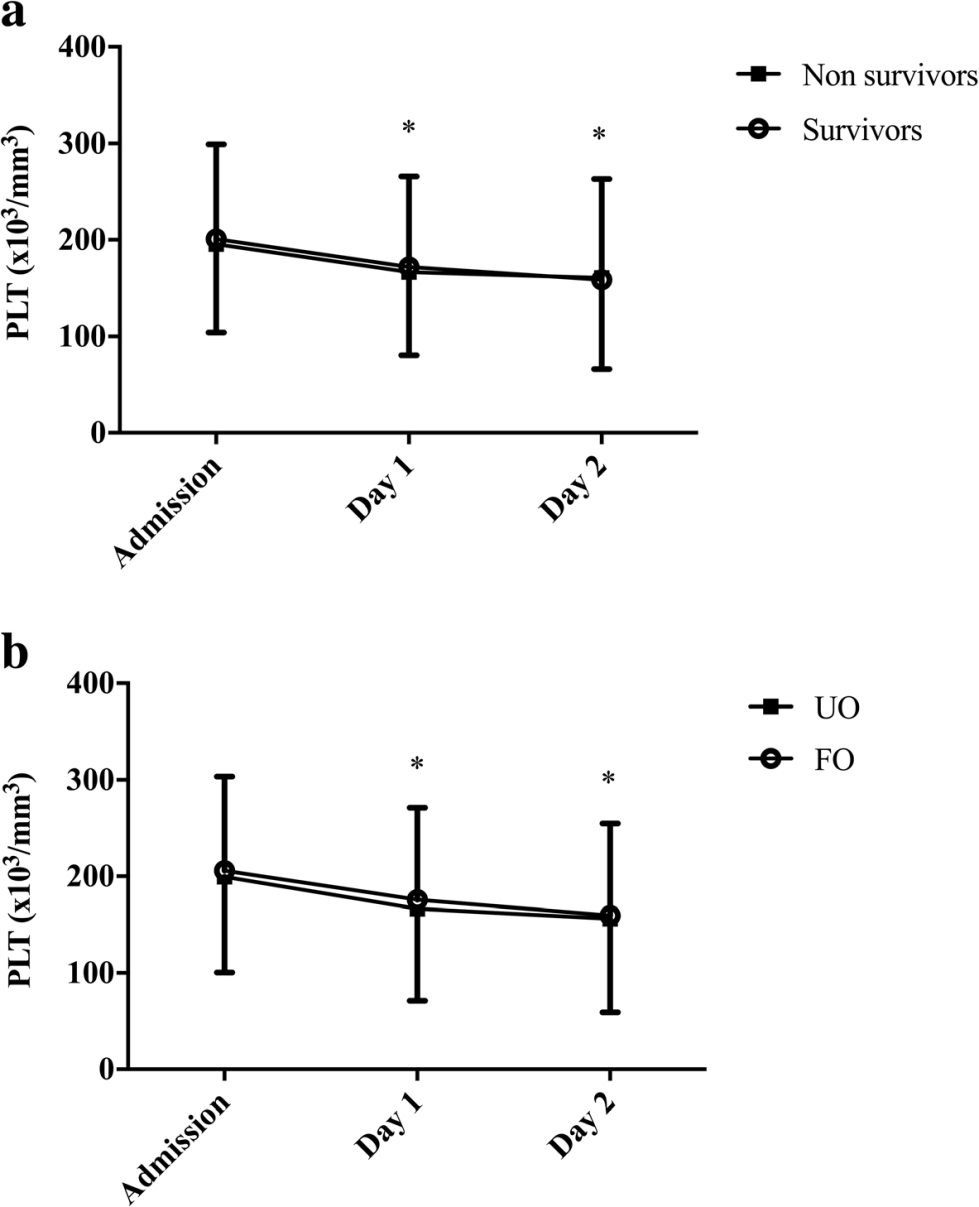
**a** Time-course of platelet count (PLT) in survivors and non-survivors and **b**) time-course of platelet count (PLT) in patients with favorable (FO) and unfavorable (UO) neurological outcome. Data are presented as mean ± SD. * = Significant difference at day 1 and 2 compared to baseline in both survivors and non-survivors, and in patients with favourable and unfavourable neurological outcome (*p* <  0.001)

**Fig. 2:**
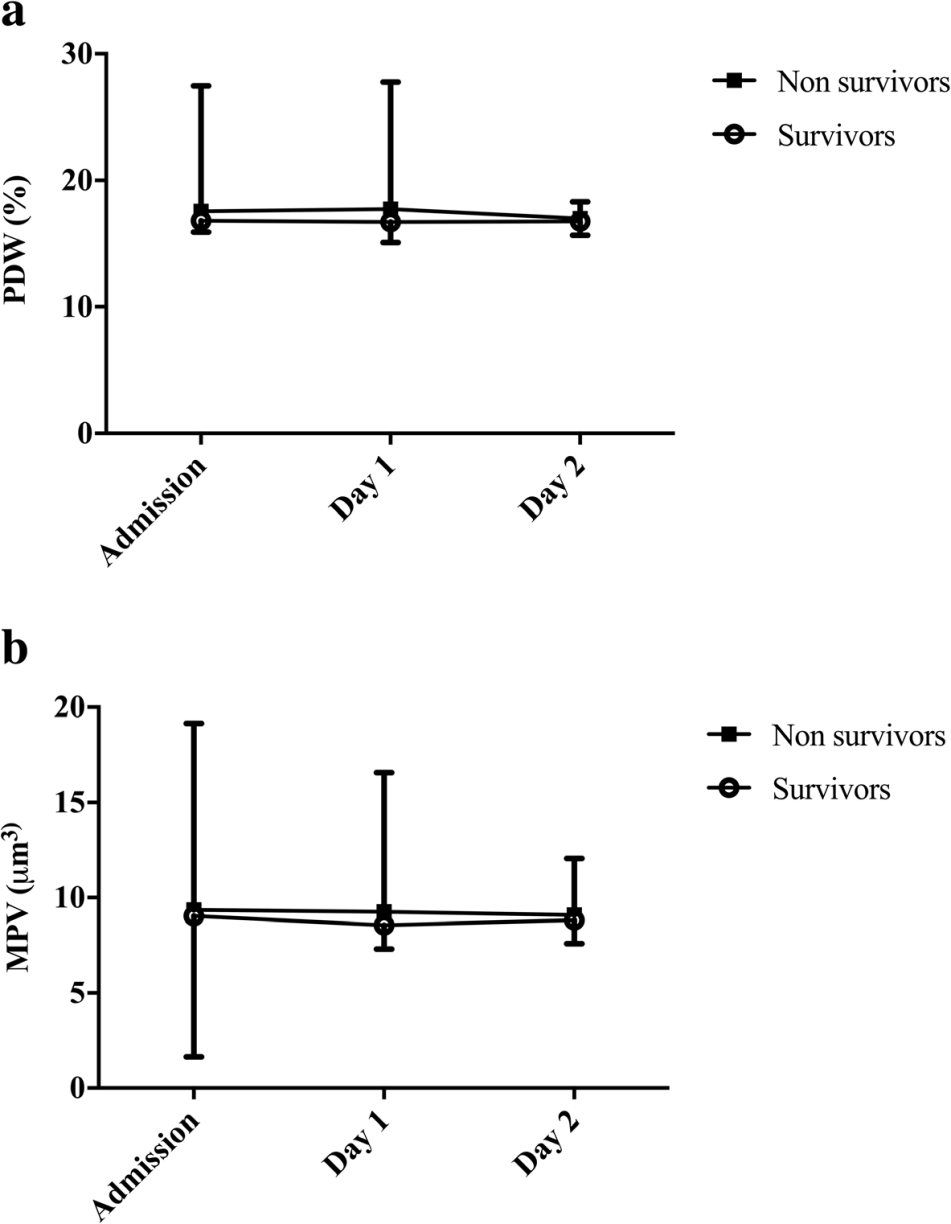
**a** Platelet distribution width (PDW) values and **b**) Mean platelet volume (MPV) in survivors and non-survivor. Data are presented as mean ± SD. Repeated measures analysis showed no difference in PDW and MPV values over time between survivors and non-survivors (*p* > 0.05)

There were no significant differences between survivors and non-survivors in admission platelet counts (196 [133–269] vs. 193 [133–261] *10^3^cells/mm^3^, *p* = 0.65), PDW (17.0 [16.2–17.3] vs. 17.0 [16.4–17.4]%, *p* = 0.42) and MPV (8.4 [7.7–9.2] vs. 8.4 [7.6–9.3] μm^3^, *p* = 0.77). The platelet count decreased significantly during the first 2 days of admission in survivors and non-survivors, whereas PDW and MPV remained stable in the two groups (Figs. 1a, 2a-b). Similar results were found when comparing patients with favourable and unfavourable outcomes (Table 1, Fig. 1b), between patients with in-hospital and out-of-hospital CA and between male and female gender (data not shown). Also, the number of patients with thrombopenia, high PDW or high MPV was similar between survivors and non-survivors or those with favourable and unfavourable neurological outcome on admission, as on day 1 and 2.

## Discussion

The platelet count decreased during the first 48 h after CA, but the PDW and MPV did not change; moreover, changes in platelet count, PDW and MPV were not associated with patient outcome.

Post-CA syndrome is associated with a decrease in the number of platelets more than an alteration in their function [2]; the etiology of the reduced platelet count may be related to a systemic inflammatory response, triggered by the ischemia/reperfusion injury occurring after the return of spontaneous circulation, and promoting the systemic activation of platelets [14]. The decrease in platelet count was unlikely to be associated with the TTM, because platelet count, activation and/or aggregation are not influenced by a moderate decrease in body temperature to 32–34 °C [15]. The prognostic role of platelet count and/or thrombopenia has been rarely described in CA patients; in one study, Nobile et al. showed no differences in the occurrence of haematological failure (i.e. platelet count < 50*10^3^cells/mm^3^) among survivors and non-survivors after CA (*n* = 469) [16], but patients with OHCA had a greater incidence of hematologic failure than those with IHCA, both on ICU admission (7% vs. 2%; *p* = 0.04) and during the ICU stay (14% vs. 7%, *p* = 0.009).

Herein, changes in PDW or MPV during could represent an important but yet unreported component of the post-CA syndrome [14]. Several studies have reported the prognostic value of PDW or MPV in critically ill patients, focusing on the fact that an increase in these variables reflects the variation in platelet size caused by platelet activation [9, 17–19]. In our study, the lack of association between these variables and outcomes may suggest that the platelet activation occurring as a result of ischemia/reperfusion injury following CA differs from that induced by sepsis or other forms of critical illness. While no study on PDW in CA patients has been published so far, Chung et al. investigated whether MPV was associated with 30-day neurologic outcome and mortality after CA. In 184 patients with OHCA, increased 30-day mortality and poor neurological outcome rate were associated with MPV on admission [20]. Further studies are needed to investigate this phenomenon by including additional tests of platelet activation and aggregation and/or thromboelastometric measurements [21, 22]. Indeed, activated platelets are enlarged, and contain vasoactive and prothrombic factors that aggravate systemic inflammation and endothelial dysfunction. Nevertheless, it is possible that PDW and MPV are not sensitive enough to detect these changes in platelet function. In one study, CA secondary to coronary occlusion had the highest degree of platelet hyperfunction, which was tested under high shear rates with the collagen adenosine diphosphate closure time, when compared to other causes of arrest [5]. This condition of platelet hyperfuncion or increased aggregability indicated a pro-coagulopathic state in the post-cardiac arrest phase, which may contribute to the formation of microthrombi (i.e. microvascular dysfunction), but also to early stent thrombosis (i.e. post-arrest acute myocardial infarction) [23]. Thus, future studies should better characterize platelet activity in order to identify patients with enhanced clot formation and who might potentially benefit from a more rapid and aggressive antiplatelet therapy.

Our study has several limitations. First, due to the retrospective design of the study, we do not have all the variables potentially influencing these platelet indices, such as the use of antiaggregants (before arrest or after hospital admission), and the presence of hematological or autoimmune diseases or a previous infectious process, in particular for in-hospital CA. Moreover, some other confounders (i.e. gender, chronic inflammatory diseases, recent trauma or surgery) may also have influenced our findings. Second, we did not specifically collect data on coagulation parameters (such as activated partial thromboplastin time or D-dimers), although these may have been altered by specific therapies (e.g., unfractioned heparin or thrombolysis) administered in CA patients. Also, the combination of intravenous and/or oral platelet drug inhibitors, which was not available for this cohort, may also have affected our results; future studies on the use of different drugs after percutaneous coronary interventions should prospectively evaluate whether PDW and MPV are affected by such interventions and whether this phenomenon would be drug- and/or dose-related. Third, we did not look at specific organ dysfunctions and their relationship with platelet count, PDW or MPV. Forth, only few patients survived with an unfavourable neurological outcome. This may reflect local practices, as decision of withdrawal of life sustaining therapies is very often shared with patients’ family in case of persistent and severe neurological impairment. Certainly, this approach prevented any additional analysis comparing survivors with good to these with poor neurological outcome. Fifth, only patients who received TTM were included in this cohort, although moderate hypothermia is not likely to influence the results. Finally, we could not record the exact time between blood sampling and laboratory analyses. In one study, Vagdatli et al. observed a remarkable decrease in PDW over time together with the absence of platelet activation, even in healthy subjects [24]; as such, samples taken outside working hours or not rapidly addressed to the laboratory for analyses may have underestimated the real PDW value and potentially biased our findings.

## Conclusions

Platelet count decreased during the first 48 h after CA, while PDW and MPV did not change. Platelets reduction, PDW and MPV were not associated with patient outcome.

## Abbreviations

CA: Cardiac arrest
CPC: Cerebral performance category score
CRP: C-reactive protein
ICU: Intensive care unit
MPV: Mean platelet volume
PDW: Platelet distribution width
ROSC: Return of spontaneous circulation
TTM: Targeted temperature management
